# Towards a new class of heavy ion doped magnetic semiconductors for room temperature applications

**DOI:** 10.1038/srep17053

**Published:** 2015-11-23

**Authors:** Juwon Lee, Nagarajan Ganapathi Subramaniam, Iwona Agnieszka Kowalik, Jawad Nisar, Jaechul Lee, Younghae Kwon, Jaechoon Lee, Taewon Kang, Xiangyang Peng, Dimitri Arvanitis, Rajeev Ahuja

**Affiliations:** 1 Quantum Functional Semiconductor Research Center (QSRC), Dongguk University, 26 Phildong 3ga, Chung gu, Seoul, 100-715, Republic of Korea; 2 Nano Information Technology Academy, Dongguk University, 26 Phildong 3ga, Chung gu, Seoul 100-715, Republic of Korea; 3 Institute of Physics, Polish Academy of Sciences, Al. Lotników 32/46, Warsaw, PL-02-668, Poland; 4 Department of Physics and Astronomy, Uppsala University, Box 520, Uppsala, SE-751 21, Sweden; 5 Applied Materials Physics, Department of Materials and Engineering, Royal Institute of Technology (KTH), Stockholm, SE-100 44, Sweden; 6 Clean Energy and Nano Convergence Center (CENCON), Hindustan University, N0.1 Rajiv Gandhi Salai (OMR), Padur (via Kelambakkam), Chennai-603103, India

## Abstract

The article presents, using Bi doped ZnO, an example of a heavy ion doped oxide semiconductor, highlighting a novel *p*-symmetry interaction of the electronic states to stabilize ferromagnetism. The study includes both *ab initio* theory and experiments, which yield clear evidence for above room temperature ferromagnetism. ZnBi_x_O_1−x_ thin films are grown using the pulsed laser deposition technique. The room temperature ferromagnetism finds its origin in the holes introduced by the Bi doping and the *p-p* coupling between Bi and the host atoms. A sizeable magnetic moment is measured by means of x-ray magnetic circular dichroism at the O *K*-edge, probing directly the spin polarization of the O(2*p*) states. This result is in agreement with the theoretical predictions and inductive magnetometry measurements. *Ab initio* calculations of the electronic and magnetic structure of ZnBi_x_O_1−x_ at various doping levels allow to trace the origin of the ferromagnetic character of this material. It appears, that the spin-orbit energy of the heavy ion Bi stabilizes the ferromagnetic phase. Thus, ZnBi_x_O_1−x_ doped with a heavy non-ferromagnetic element, such as Bi, is a credible example of a candidate material for a new class of compounds for spintronics applications, based on the spin polarization of the *p* states.

Dilute magnetic semiconductors (DMS) are promising materials for spintronics applications as they exploit the intrinsic spin of the electron in addition to the electron charge. Above room temperature ferromagnetism has been reported in nitride and oxide semiconductors such as GaN and ZnO^1^,^2^,^3^,^4^. There are many theoretical^5^,^6^ and experimental^7^,^8^,^9^ studies on DMS in which the cations are partially substituted with transition metal (TM) ions to achieve ferromagnetism above room temperature. However, an undesirable phase separation can occur and constitutes a problem in the TM doped DMS, often hindering its practical applications^10^. Moreover, transition metal oxide inclusions may be ferromagnetic themselves, giving rise to doubts on the origin of ferromagnetism in transition metal doped oxide and nitride semiconductors and causing complications for the growth of practical DMS materials. We can minimize the problem of the formation of magnetic precipitations by doping with a non ferro- or non ferrimagnetic element in its bulk and oxide forms. If the dopant and its oxides are non ferro- or ferrimagnetic in their bulk form, formation of precipitates or clusters is less likely to contribute to the ferromagnetic response of a DMS based material. Recently, it was found that DMS with Curie temperature, *T*_*C*_, above 300 K can be synthesized by doping with non-TM atoms to substitute for the anions in the semiconductors. It is observed that the generation of holes in the system can stabilize ferromagnetism in the ground state of a DMS material^11^. Ferromagnetic stability of non-TM doped systems depends on the concentration of holes and the exchange splitting of the dopant gap states^11^,^12^. It is also demonstrated that ferromagnetism could originate within a semiconductor by means of the introduction of free carriers in the system, using anion doping, such as N and C^13^,^14^. Focusing on non-TM ions as doping elements, in an itinerant picture of the electronic structure, the choice of heavy non-magnetic elements presents the advantage of stabilizing the ferromagnetic response due to the stronger spin-orbit energy associated with the ion cores of heavy atoms, linking the magnetic moments with the lattice. It also allows to highlight the impact of electronic states other than the ones of *d*-symmetry on the mediation of the ferromagnetic interaction. A *p*-element doping by means of lighter atoms, as for example B in ZnO, leads also to a ferromagnetic phase of this material, however in this case the occurrence of ferromagnetism is related to defect formation^15^. In contrast, doping with a heavy *p* element, such as Bi, leads to *p-p* related itinerant ferromagnetism, not defect related. This finding strongly suggests that the spin-orbit coupling is indeed a key component to favour itinerant magnetism in ZnO. To our knowledge, this article is the first report on ferromagnetism in a *p*-metal doped semiconductor. It is well known that doping with a heavy element leads to an increase of the magnetic anisotropy and ultimately the magnetisation for metallic systems, even in the presence of disorder. Thus, non-TM and non-ferromagnetic heavy ion doped DMS materials may yield a radically new direction in the search for novel spintronic materials.

As a practical example of this new general approach, we focus here on a detailed experimental and theoretical study of the heavy atom dopant, bismuth, which has an explicit impact on the electronic states of *p*-symmetry in the semiconductor matrix and the mediation of the ferromagnetic interactions. In this new class of DMS materials, magnetic properties are explained by the *p*-orbital interaction between the dopant and the host atoms. Bismuth doping in ZnO has been carried out by a few groups in order to study varistors^16^, and Bi induced acceptor states^17^,^18^. Our recent study on Bi-doped ZnO shows a stable *p*-type conductivity of the films^19^. To our knowledge none of the groups so far have explored the existence of a ferromagnetic phase in Bi doped ZnO and its atomic origin. Bismuth in perovskites is known to induce ferromagnetism in materials like BiMnO_3_ and BiFeO_3_^20^,^21^. In this work we present experimental and theoretical evidence of room temperature ferromagnetism of Bi doped ZnO and we explore its microscopic origin in this system. We establish that the exchange interaction is mediated by means of the holes generated by anionic doping. We use a combination of traditional inductive magnetometry, with element specific x-ray magnetic circular dichroism to probe the magnetic response of the full sample and the elemental magnetic response of the O atoms both in the surface region and in the bulk of the ZnO films.

## Experimental and Theoretical Results on Bi Doped ZnO Thin Films

### SQUID magnetonetry

**Figure 1:**
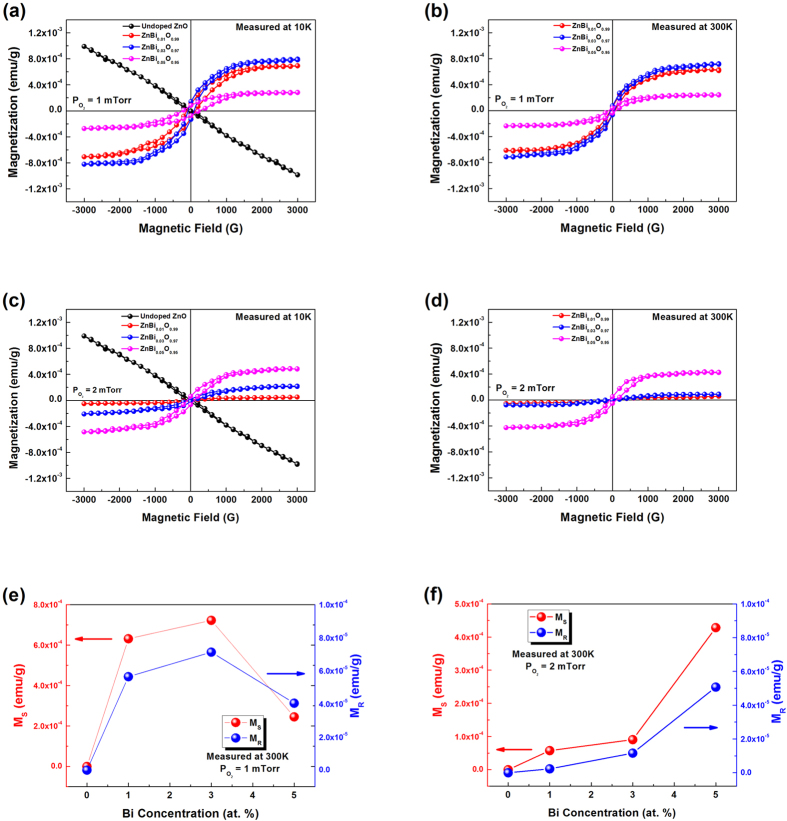
Magnetization versus magnetic field curves are shown for ZnBi_x_O_1−x_ thin film samples grown under a 1 mTorr O_2_ pressure, measured at (**a**) 10 K and (**b**) 300 K using SQUID magnetometry. For ZnBi_x_O_1−x_ thin film samples grown under a 2 mTorr O_2_ pressure measurements are shown at (**c**) 10 K and (**d**) 300 K. The saturation (**e**) and the remanent magnetization (**f**) are shown versus bismuth concentration at 300 K for the samples grown under a 1 mTorr and 2 mTorr O_2_ pressure, respectively.

In order to determine the magnetization of the ZnBi_x_O_1−x_ thin films we perform inductive magnetometry experiments. Figure 1(a–d) show the magnetization versus magnetic field (M-H) curves measured for ZnBi_0.01_O_0.99,_ ZnBi_0.03_O_0.97_ and ZnBi_0.05_O_0.95_ thin films (grown at 1 mTorr and 2 mTorr of O_2_ pressure as indicated in Fig. 1) at the temperatures of 10 K and 300 K. The M-H curves for the undoped ZnO epilayer give clear proof that the ZnO film is not ferromagnetic as seen in Fig. 1(a,c). In order to rule out the defect related ferromagnetism phenomenon in undoped ZnO epilayers, we carried out magnetisation measurements on undoped ZnO films grown at various oxygen partial pressures. These undoped films turned out to be diamagnetic. We can deduce for the doped ZnO a change in the sign of the magnetic susceptibility versus the undoped case. The M-H curves presented in Fig. 1(a–d) indicate that the ZnBi_x_O_1−x_ thin films are ferromagnetic at room temperature.

Direct evidence for the incorporation of Bi into the ZnO lattice is provided by the SIMS data (Fig. S2). Further details on the study of Bi incorporation in the ZnO lattice as well as the sample growth and composition have been published earlier^19^. Furthermore, the clear opening of the M-H curves indicate long range ferromagnetism in ZnBi_x_O_1−x_ thin films. Fig. 1(e,f) depict the saturation magnetization and the remanent magnetization as a function of Bi concentration in the ZnBi_x_O_1−x_ thin films, grown at 1 mTorr and 2 mTorr of O_2_ pressure and measured at 300 K. This specific plot allows to quantify the magnetization of the ZnBi_x_O_1−x_ films at room temperature. The value of the remanent magnetization is highest for the ZnBi_0.03_O_0.97_ thin film grown at 1mTorr of O_2_ pressure. A maximum is found for the saturation magnetization for samples grown at 1mTorr O_2_ pressure around a 3% Bi concentration. The remanent magnetization and coercive field (H_c_) of the ZnBi_0.03_O_0.97_ sample measured at 10 K are 1.419 × 10^−4^ emu/g and 85.3 G. At 300 K these values are 7.218 × 10^−5^ emu/g and 61.4 G, respectively. The magnetization values for the ZnBi_0.03_O_0.97_ sample are highest as it is also evidenced by our theoretical calculations. The magnetization measurements are complemented by means of x-ray absorption and x-ray magnetic circular dichroism at the O K-edge. By using energy tunable circular x-rays at a synchrotron radiation laboratory, we probe the spin polarization of the O(2*p*) states only. The magnetic moments obtained for the O atoms are in agreement with the theoretical values and with the magnetization measurements.

### Density functional theory calculations

The calculated lattice parameters for wurtzite ZnO are found to be a = 3.28 Å and c/a = 1.617 within the Generalized Gradient Approximation (GGA) framework. These values are in good agreement with experimental data^22^. The relative energetic stability of Bi doped ZnO can be obtained from the calculations based on Density Functional Theory (DFT). The formation energy^23^ of ZnBi_x_O_1−x_ can be defined as follows:


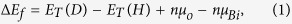


Here 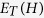 and 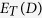 are the total energies of pure and doped ZnO, respectively. The term *n* is the number of the doping atoms. 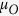 and 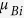 are the atomic potentials of the oxygen and Bi atoms, respectively. The formation energy of the ZnBi_x_O_1−x_ with respect to the bulk and isolated Bi atom is given in Table 1.

**Figure 2:**
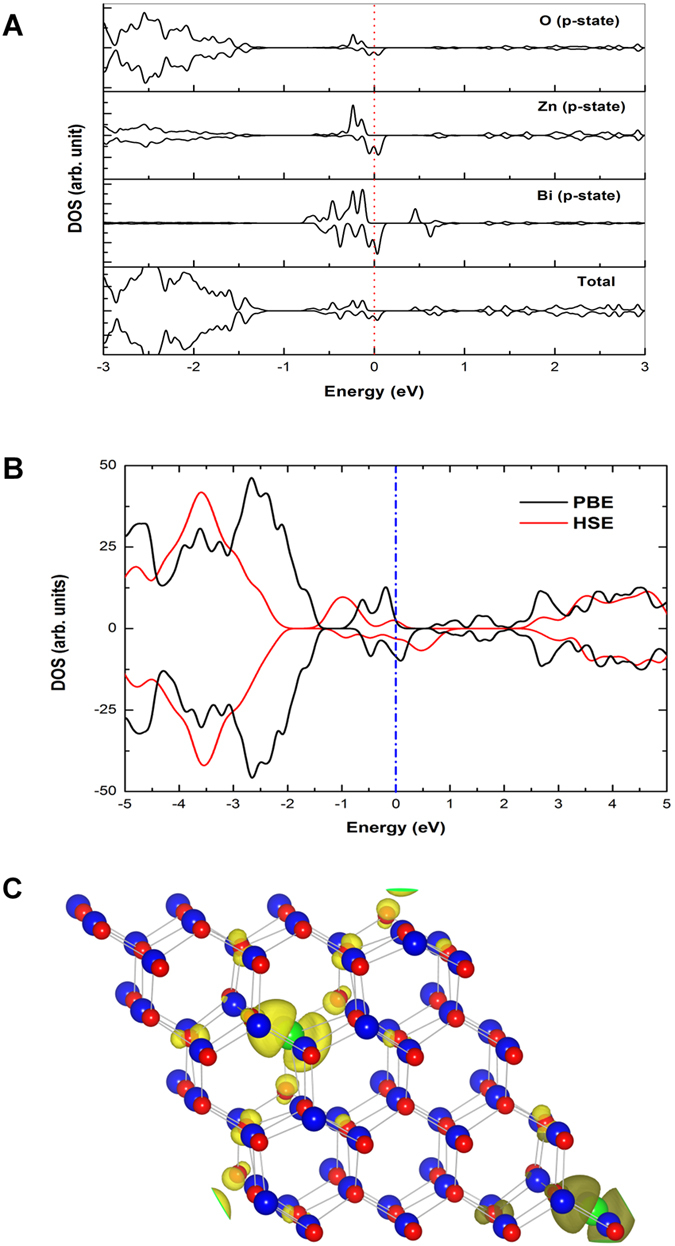
The total and partial density of states of ZnBi_x_O_1−x_ (x = 3.12%) of ferromagnetic alignment are shown in (**a**), the vertical red dotted line represents the Fermi energy. The total density of states (TDOS) of ZnBi_x_O_1−x_ (x = 5.5%) by GGA-PBE and HSE are shown in (**b**), the vertical blue dashed line indicates the Fermi level. The calculated spin charge density (ρ↑–ρ↓) in the Bi-doped ZnO (3.1%) is shown in (**c**). Bi, Zn and O are represented by green, blue and red spheres, respectively.

To study the magnetic properties of this compound, Bi atoms are placed in two oxygen sites in the supercell, for different doping concentrations. The estimated formation energies of the system are 0.06 and 0.11 eV per formula unit at the doping level of 1.56% and 3.12% respectively, versus bulk ZnO and an isolated Bi atom. In the case of ZnBi_x_O_1−x_ with a doping concentration 3.12%, our calculations show that the system is ferromagnetically stable with the energy difference of 108.5 meV (Table 1). The total magnetic moment of the system is 1.25 μ_B_. The nearest Zn atoms and the second nearest oxygen atom also contribute to the magnetic moment by small amounts (0.033 μ_B_ and 0.022 μ_B_, respectively). We have calculated the spin polarized total and partial density of states (PDOS) of the *p*-states of bismuth as well as its neighbouring Zn and oxygen atoms in the system with the doping of 3.12%, which is shown in Fig. 2(a). For the doping concentration of Bi of 1.56%, the system is again ferromagnetic with the energy difference of 17 meV (Table 1). The observed magnetic moment in ZnBi_x_O_1−x_ is 0.15 μ_B_ per Bi atom with 1.56% of bismuth doping. It is observed that the magnetic moment per Bi atom is increased with the increase of doping concentration in the ZnBi_x_O_1−x_.

The experimental results agree well with the theoretical findings. It is also interesting to note that the various oxide phases of bismuth are not ferromagnetic, strongly suggesting that the ferromagnetic phenomenon in the Bi doped ZnO is due to bismuth substitution in the ZnO lattice.

As there is an anionic substitution in oxygen sites, our theoretical study predicts ferromagnetism due to *p-p* interaction between the bismuth and oxygen atoms. Moreover, the substitution of bismuth in the oxygen sites is known to create holes. As the bismuth doped ZnO epilayers exhibit a *p*-type conductivity, it is clear that holes are created during bismuth substitution in oxygen sites. Despite the fact that some of the compositions of bismuth had unstable *p*-type properties, overall, holes play a role in the ferromagnetic coupling, at least as minority carriers. Studies on bismuth induced acceptor states carried out elsewhere^16^ support our argument. Our DFT calculations predict ferromagnetism in the system where bismuth is substituted at the oxygen site of ZnO with various doping concentrations. The energy difference between parallel and antiparallel alignment in Bi doped ZnO is lower than Mn doped ZnO^24^, but still this energy difference is sufficient to justify room temperature ferromagnetism. The main contribution to the magnetic moment in the system is due to the *p*-orbitals of bismuth with 0.26 μ_B_ per atom. The magnetic moment per Bi atom increases with increasing doping concentration as the distance between the dopant and the neighbouring O atoms decreases on average and exchange coupling becomes stronger.

The ferromagnetism in ZnBi_x_O_1−x_ is due to the *p-p* interaction between the dopants and host atoms instead of *p-d* and *d-d* interactions which have been considered earlier^13^,^25^. When the Bi atom occupies the substitutional site of oxygen, it leads to the creation of holes in the electronic structure. The local magnetic moment of Bi in ZnO is due to the relatively localized Bi p-states and the charge transfer from the neighbouring Zn atoms. The Zn and O atoms which are located closest to the Bi atoms are also slightly spin-polarized (Fig. 2(a)). In order to take into account the effects of nonlocal exchange in Bi-doped ZnO, we apply the Heyd-Scuseria-Ernzerhof (HSE) hybrid functionals^26^. Figure 2(b) shows the total density of states of Bi doped ZnO. To visualize the atomic distribution of spins in the Bi doped ZnO, we show the spin charge density in Fig. 2(c). These results clearly indicate that the induced magnetic moments are mainly contributed by the Bi atoms (0.26 *μ*_B_/atom), while the neighbouring oxygen atoms carry very small magnetic moments (less than 0.05 *μ*_B_). The Fugure 2(c) also shows that the spin-polarized electrons are much localized at the Bi atoms. We have also worked beyond the GGA framework by employing the GGA + *U* approach. First, we have tried for Hubbard *U* values of 5, 6, 7 and 8 eV on the Bi 5*d* states in the Bi-doped ZnO system; there is no effect of U on the density of states because the 5*d* electrons of Bi are very localized. We have also applied the Hubbard *U* correction on the Zn 3*d* atoms. The Zn 3*d* states are located well below the Fermi energy so there is little effect on the energy gap and the valence band is pushed below by 0.4 eV. We cannot find any change in the density of states at the Fermi level using the Hubbard *U* correction. The *p-p* interaction in anion doped DMS has a long range character as it is found that ZnBi_x_O_1−x_ is still ferromagnetic with a doping concentration as low as 1.56%. We have tried to find other possible sources for ferromagnetism by introducing O-vacancies in Zn_1−x_Bi_x_O, Bi-interstitials in ZnO and Zn-interstitials in Zn_1−x_Bi_x_O, but our calculations show that these systems are non-ferromagnetic.

## Element Specific Magnetometry

### Experimental XAS and XMCD results

**Figure 3:**
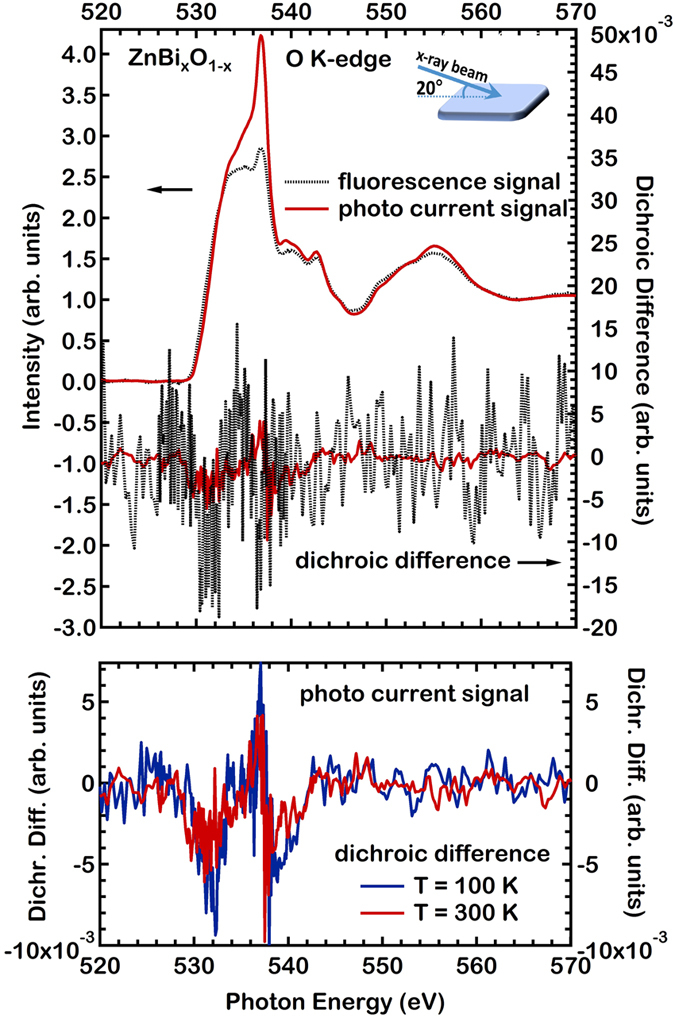
Experimental XAS and XMCD spectra are shown, taken with close to circular x-rays for a ZnBi_x_O_1−x_ (x = 4%) thin film. Grazing x-rays are used at 20° from the surface plane. The dichroic (XMCD) signal is taken by reverting the applied magnetic field. The XMCD difference is stronger for TFY.

Given the previous results, we employ x-ray absorption spectroscopy to characterize the magnetism of ZnBi_x_O_1−x_ films grown by pulsed laser deposition at the atomic level. To probe for O(2*p*) electron related magnetism linked with the Bi doping, we use circular x-rays at a synchrotron radiation laboratory^27^ to excite the O(1*s*) core electrons, leading to dipole transitions to the O(2*p*) states. To revert the magnetic moment of the O atoms we apply a magnetic field *in situ*. In Fig. 3 O *K*-edge XAS spectra are shown, taken with circular x-rays. The characteristic intensity variations close to the O *K*-edge, are due to transitions to O(2*p*) final states. The observation of O *K*-edge XMCD provides direct evidence that the O atoms carry a magnetic moment. We observe that the Total Electron Yeild (TEY) and Total Fluorescence Yield (TFY) lead to strong differences in spectral features as observed earlier in ZnO thin films^28^. Differences in the TEY versus TFY spectra highlight the possibility for a different electronic and real space structure of the near surface region of the film versus the film interior. In general, *K*-edges lead to rather small XMCD effects. Nevertheless, we observe a clear XMCD response from the O atoms under the applied magnetic field of 0.5 T. The XMCD response at the O *K*-edge highlights that the O(2*p*) states carry considerable spin polarization, in agreement with the previous *ab initio* theory results and the magnetization measurements.

### *Ab initio* calculations of x-ray absorption

**Figure 4:**
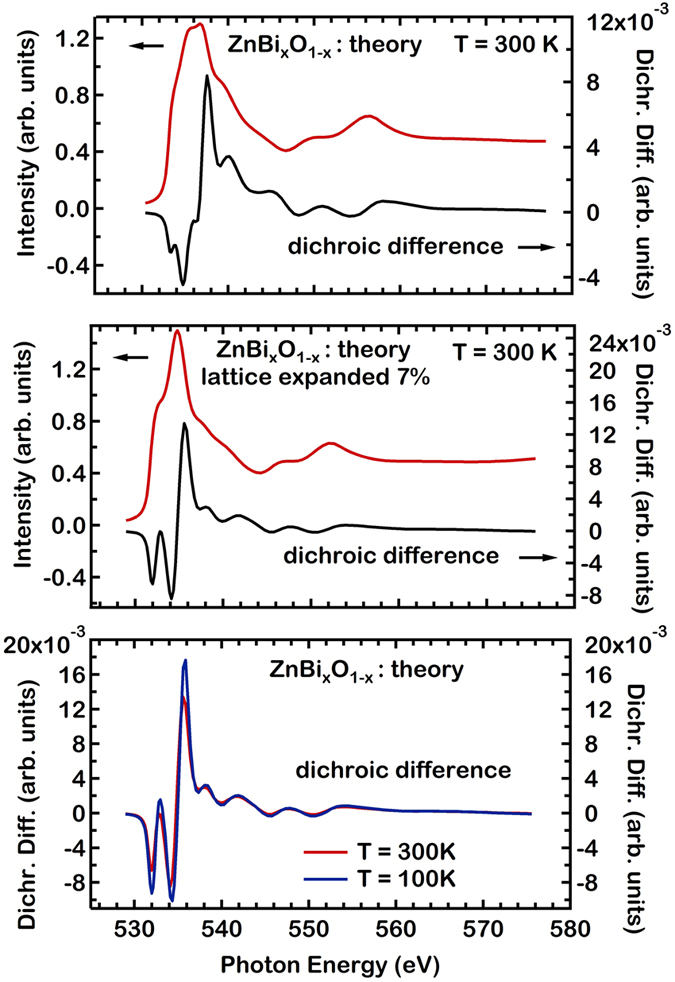
Theoretical XAS and XMCD spectra are shown for ZnBi_x_O_1−x_ (x = 3%) which are calculated with the FEFF code. Close to circular light is used, with the same degree of circularity as for the experiment. The Bi doped ZnO spectra using the known bulk values for the ZnO lattice describe well the experimental TFY spectra. The TEY spectra, are best described, by means of an expanded ZnO lattice, here a calculation with an expansion of 7% is shown. The TEY channel is sensitive to the near surface region of the ZnBi_x_O_1−x_ film, only with TFY the bulk of the ZnBi_x_O_1−x_ film is probed. The thermal disorder on the XAS and XMCD spectra is introduced using the correlated Debye model.

In order to gain further quantitative insight into our experimental XAS and XMCD spectra, we compare our data with theoretical spectra. We use the FEFF code, which is an *ab initio*, self-consistent, multiple scattering code for the simultaneous calculations of excitation spectra and electronic structure^29^,^30^. Here we work with the real space option of the FEFF code, suitable for precise calculations also at higher energies above the edge. We discuss a wider energy range (529–570 eV) for both XAS and XMCD spectra, given the intensity variations observed in our experimental results. We now turn to the results of our FEFF calculations for the XAS ZnBi_x_O_1−x_ shown in Fig. 4. To simplify the claculation, the ZnO crystalline structure is used, without static disorder or defects. To obtain the characteristic shape of the XAS features in the TEY spectra where the signal to noise ratio is much higher, a lattice expansion is found necessary. A volume lattice expansion, for the surface region, by 8(2)% yields much beter agreement to the TEY spectra. The TFY spectrum appears to be consistent with a mixture of a spectrum corresponding to the bulk value of the ZnO lattice together with spectra corresponding to an expanded lattice. The Bi atoms are randomly substituted to 3% at the O sites, for these claculations. The theoretical spectra indicate that the wurzite hexagonal plane for the ZnO lattice, lies within the surface plane of the sample. The dominant spectral features seen in the experiment can be reproduced by the theory, both in terms of energy and relative intensity, if the existence of a lattice relaxation is assumed for the near surface region probed by TEY.

We now turn to the possibility of the FEFF code, for the calculation of XMCD difference spectra. Focusing on the origin of the XMCD signal at the O *K*-edge we present results of two limiting cases relating with the present experimental material. We calculate the XMCD of a volume expanded ZnO lattice by 7% to model the film surface region probed by TEY and also the bulk ZnO structure, a contribution to the signal which should be present in the TFY results. The data of Fig. 4 show theoretical ZnBi_x_O_1−x_ XMCD difference spectra for the expanded ZnO lattice. The spin is reversed and the two obtained spectra are then subtracted to obtain the XMCD difference shown in Fig. 4. The spin values used for the O, Zn and Bi atoms are the ones obtained from *ab initio* theory, namely for the O atoms a spin moment of 0.022 μ_Β_/atom, for Zn 0.033 μ_Β_/atom and for Bi 0.26 μ_Β_/atom.

The result of the spin dependent calculations of Fig. 4 reproduce fairly well the strength of the experimental XMCD difference in Fig. 3 for grazing x-ray incidence. The experimental O *K*-edge XMCD difference consists of oscillations versus the photon energy, strong very close to the edge, which become weaker as the photon energy increases. Also the calculated XMCD consists of fast oscillations. We find the amplitude of the calculated XMCD signal to be within a factor of 2 from the one observed in the experiment in Fig. 3 (right Figure axis), using the theoretical magnetic moments for the Zn, O and Bi atoms. The XMCD signal, as predicted earlier by theory, starts at the absorption edge by transitions to delocalized final states exhibiting O(2*p*) character. This appears to be a general feature of Bi doped ZnO. Expanding the lattice and superimposing XMCD oscillations with slightly diffferent phase for each lattice value, leads then to average XMCD oscillations which may tend to cancel out with increasing photoelectron kinetic energy, as observed in the experiment. This is due to the fact that these oscillations start in phase at the absortion edge but have a different phase, leading to destructive interference at higher photoelectron kinetic energies. The discrepancy of the theoretical XMCD signal versus the experimental one, may be also due to the existence of structural disorder, in particulat for the TEY in the near surface region. Also the magnetic response for various ZnO lattice constants is varying strongly, as shown in Fig. 4. The XMCD signal for the TFY channel, will be also a superposition of several contributions due to the finite probing depth, still tending to lower the overall signal amplitude. Overall the fact that the *ab initio* FEFF code reproduces several characteristics of the XMCD signal, stongly supports the values of the magnetic moments as determined by the theory. We have been able to identify a plausible reason for the discrepancy of the XAS data between the TEY and TFY channels, namely an expansion of the lattice. Modelling of the XMCD would require not only the use of the *ab initio* FEFF code for a variety of geometries, but also a weighted average of these results using the TEY probing depth into the surface, taking into account the roughness of the surface, increasing the number of free parameters. The FEFF results describe correctly a negative dichroic signal directly at the absorption edge. They fail however to yield a negative signal at higher energies. Possible reasons for this discrepancy, beyond structural disorder and surface relaxation, may lie in the presence of an orbital moment contribution, which is not addressed here. The possible presence of an orbital moment is consistent with the strong spin orbit energy of the Bi atoms.

## Conclusion

In summary, our experimental findings and *ab initio* calculations reveal that Bi doped ZnO is a potential prototype system for a novel class of dilute magnetic semiconductor materials, which are ferromagnetic at room temperature Free carriers in the system are found to play an important role in the electronic and magnetic properties of anion doped DMS. The origin of ferromagnetism in ZnBi_x_O_1−x_ can be traced to the *p-p* coupling interaction between Bi, Zn and O atoms. Doping with Bi, a heavy *p* element, leads to itinerant ferromagnetism which is not defect related. In contrast, doping with B, a light *p* element, does not lead to a stable itinerant ferromagnetic phase. Our findings strongly suggest that the *p-p* type of interaction, in combination with the spin-orbit coupling, are key ingredients the doping atom has to introduce into the ZnO lattice, to stabilize itinerant ferromagnetism. Our results are valid for materials such as GaAs, GaN and TiO_2_. Further efforts should be put forward, using heavy doping elements which favour a *p*-symmetry coupling, and stabilize itinerant ferromagnetism through enhanced spin-orbit coupling.

## Experimental Section

### Sample fabrication and experimental details

The bismuth doped ZnO epilayers are grown on a (0001) sapphire substrate using a fully automated ultra high vacuum pulsed laser deposition (Neocera, Pioneer 180, Beltsville, VA, USA). The bismuth doped ZnO target is prepared by a conventional solid state reaction at 1000 °C using Bi_2_O_3_ (99.999%) and ZnO (99.999%) powders as precursors. The ZnBi_x_O_1−x_ films are deposited at 600 °C under an oxygen pressure of 1 mTorr and 2 mTorr, using an excimer laser with a wavelength of 248 nm, operating at 5 Hz with a fluence of 1.5 J cm^−2^. Also an undoped ZnO epilayer is grown under the conditions mentioned above to compare with our results on the ZnO thin films doped with bismuth. The thickness of the films is close to 200 nm in all cases. X-Ray Diffraction (XRD) studies are carried out using a Rigaku (Model: XRD-DMAX Rapid) x-ray diffractometer to characterise the crystallinity and growth orientation. X-ray Photoelectron Spectroscopy (XPS) is used to confirm the existence of bismuth in the ZnO films using the VG Microtech (Model: ESCA-2000) ESCA station. A Superconducting Quantum Interference Device (SQUID) is used for the magnetization measurements using the MPMS (Magnetic Property Measurement System)-XL system.

X-ray Absorption Spectroscopy (XAS) and X-ray Magnetic Circular Dichroism (XMCD) measurements are carried out at beam line I1011 of the MAX-lab synchrotron radiation laboratory, Lund,Sweden^27^. The Elliptically Polarizing Undulator x-ray source of this beam line allows to take XAS spectra with soft x-rays of a polarization state both close to circular or linear. The measurements are performed in the Total Electron Yield (TEY) mode by measuring the photocurrent of the sample. In the TEY mode the effective mean free path of the electrons is of the order of 2 nm, implying that only a region down to 6 nm below the sample surface is probed in this mode. Also the Total Fluorescence Yield (TFY) is recorded simultenously using a soft x-ray photodiode based detector, allowing to probe also the bulk of the ZnO films, given the longer escape length of the soft x-ray light at the energies considered here, of order 100 nm. The samples are introduced into a Ultra High Vacuum chamber, equipped with eight magnetic coils, allowing for a magnetic field of 0.5 T to be applied in any space direction (Soft X-ray Scattering Octupole End Station from Advanced Design Consulting). The soft x-ray range between 0.2 and 1.1 keV is used to characterize the chemical composition of the samples. The state of the sample near surface region did not necessitate any ion sputtering for cleaning purposes. The angle dependence of the XAS for the ZnBi_x_O_1−x_ films is investigated by rotating the sample around the polar axis, taking XAS spectra between 90° normal and 15° grazing x-ray incidence. For the XMCD experiments the sample is positioned at a 15° x-ray incidence angle with the magnetic field along the x-ray propagation direction. The XMCD difference is obtained by reverting the applied magnetic field and keeping the helicity of the x-rays constant. For the angle dependent XAS and XMCD linear and close to circular polarization (with a degree of circular polarization of 0.90(5)) is used, respectively.

### Computational Details

To verify our experimental findings the first-principles calculations are carried out using the projected augmented wave (PAW) method^31^, as implemented in the Vienna *ab initio* Simulation Package (VASP)^32^,^33^,^34^. In the study of the magnetic order, two O atoms are substituted by Bi atoms in the 4 × 4 × 4, 4 × 4 × 2 and 3 × 3 × 2 supercell, corresponding to an effective Bi-concentration of 1.56%, 3.12% and 5.56% respectively, which are comparable with the experimental doping level. We carried out an optimization of the geometry of the ZnO system before and after the various dopings. The Generalized Gradient Approximation (GGA-PBE)^35^ is used for the exchange-correlation potential. The plane-wave cutoff energy in our calculations is set to 400 eV. The atomic geometries are fully optimized until the forces on each atom are less than the threshold value of 10^−5^ eV/Å. The valence states of the potentials of Zn, Bi and O are 3*d*^10^3*p*^2^, 6*s*^2^6*p*^3^ and 2*s*^2^2*p*^4^, respectively. The Gaussian smearing width is 0.2 eV. Brillouin zone integrations are performed with a Gamma centered 2 × 2 × 2 mesh^36^. ZnO has the hexagonal wurtzite structure with space group P6_3_mc (186) at ambient conditions.

To calculate the XAS spectra we use the FEFF8.4 version of the FEFF code and some of the features of its FEFF9 version. We use the possibilities of this code, for the calculation of the spectra of clusters of atoms^29^,^30^. We have used earlier the FEFF code to calculate the N K-edge XAS of GaN^37^, our results for GaN are consistent with earlier FEFF calculations^38^. A ZnO cluster with a radius of 12 Å (608 atoms) is used, around the photo-excited O atom, for the calculations in the wurzite crystal structure, with lattice constants of 3.249 (*x*, *y* axis of the hexagonal unit cell in the hexagonal plane), 5.207 Å (*z* axis along the hexagonal axis) and the parameter *u* = 0.375. A Hedin-Lundqvist energy dependent exchange correlation potential with a constant imaginary part is used following earlier calculations done with the FEFF code^29^,^30^. An imaginary part of 0.25 eV is used here. No other broadening factors are used to match the experimental data. This value falls within the typical value range used earlier for this type of potential^29^,^30^. Systematic calculations are performed, varying the radius of the atom cluster around the photo-excited atom, for the use of self consistent potential calculations and for full multiple scattering. To obtain convergence of the spectral features close to the absorption edge, the first few eV, at least a radius of 8 Å (177 atoms) are found necessary for self consistent potentials and for full multiple scattering. The calculations shown here are performed with self-consistent potentials at 10 Å (336 atoms). The core hole is modelled using the final state rule option in the code. A screened core hole (FEFF9) was also tried but only small differences in intensities of spectral features were observed. The overall agreement versus the experimental spectra obtained that way is not much better overall, if the first 10 eV close to the edge are considered. We show here the results using the final state rule option available already at the FEFF8.4 version of the code. As the measurements are done at 100 K and 300 K, to describe thermal effects the correlated Debye model is used with a Debye temperature of 400 K, the known bulk Debye temperature of ZnO.

The FEFF code as used here presents the advantage of calculating the full absorption spectrum, including the continuum step function. This allows to perform a quantitative analysis of the angular, or XMCD magnetic response on a per atom basis, as for the experimental spectra, without additional adjustable parameters, as the high energy continuum can be used to normalize the spectral response on a per atom basis in the same manner as for the experimental spectra. Both the 8.4 and 9 versions of the FEFF code allow to include spin polarization in the calculations, assigning a spin value to a specific atom. The spin-dependent potentials are calculated from the spin-dependent densities to construct the spin-dependent muffin-tin potential^29^,^30^,^39^. In particular the XMCD spectra of Ni, Fe and Gd were satisfactorily reproduced using the FEFF8 code^39^,^40^. A straightforward over-simplified “free atom like” interpretation of the XMCD signal based on a measure of the spectral areas in combination with sum rules, to determine an orbital moment contribution, is challenging due to the fact that we deal with O atoms in the neighbourhood of Bi atoms in the ZnO lattice. For a core hole excitation for the O atoms exhibiting hybridization with the Bi atoms, the strong spin orbit energy of the Bi atoms is expected to lead to a breakdown of a simplified analysis not allowing for a reliable determination of the magnetic moment based on sum rules. Deviations from the simple **LS** coupling scheme have already been reported from XMCD work in the literature, in the vicinity of heavy atoms in the bulk^41^ (in EuO) or at interfaces^42^ (in Fe/Wand Fe/Ir multilayers) making the use of theory to model the XMCD signal the most reliable way to obtain an estimate of the magnetic moments using XMCD.

## Additional Information

**How to cite this article**: Lee, J. *et al.* Towards a new class of heavy ion doped magnetic semiconductors for room temperature applications. *Sci. Rep.* **5**, 17053; doi: 10.1038/srep17053 (2015).

## Supplementary Material

Supplementary Information

## Figures and Tables

**Table 1:** The formation energy, the magnetic moment per Bi atom for different doping concentration in the ZnBi_x_O_1−x_ system and the energy difference between antiferromagnetic and ferromagnetic alignments are given.

Zn Bi_x_O_1−x_	Formation Energy (eV/f.u)	Magnetic moment/Bi atom (μ_B_)	ΔE = E_AFM_–E_FM_ (meV)	Stability
x = 0.015	−0.06	0.15	17.3	FM
x = 0.031	−0.11	0.26	108.5	FM
x = 0.055	−0.18	0.45	65.5	FM

For all the concentrations shown here the ferromagnetic alignment is found as the most energetically stable.
