# Supplementary material for: Towards a new class of heavy ion doped magnetic semiconductors for room temperature applications
Source: Sci Rep. 2015 Nov 23;5:17053. doi: 10.1038/srep17053 (PMC4655408; doi:10.1038/srep17053)
Supplement: Supplementary Information [file srep17053-s1.pdf]

Supporting Information

Towards a new class of heavy ion doped magnetic semiconductors for room temperature applications

Juwon Lee, Nagarajan Ganapathi Subramaniam, Iwona Agnieszka Kowalik, Jawad Nisar, Jaechul Lee, Younghae Kwon, Jaechoon Lee, Taewon Kang, Xiangyang Peng, Dimitri Arvanitis, Rajeev Ahuja*

Experimental Details:

Bismuth doped ZnO epilayers were grown using Ultra High Vacuum Pulsed Laser Deposition system(UHV-PLD) from Neocera LLc. Homogenous targets of $ZnBi_xO_{1-x}$ with bismuth concentration x=1 at.%, 3 at.% and 5at.% were prepared by thorough mixing and sintering of ZnO(99.999%) and Bi_2O_3 (99.999%) powders at 1000 °C. The base pressure of the chamber was 2×10^{-8} Torr. The growth was carried out at 1×10^{-3} Torr. and 2×10^{-3} Torr. With the flow of high purity oxygen. Epilayers were deposited on a cleaned sapphire (0001) substrate using a KrF excimer laser of 248 nm wavelength operating at 5 Hz and with a fluence of 1.5 J cm^{-2}. The growth temperature was maintained at 600 °C. The PLD system was a fully automated one so that the growth parameters were controlled with high accuracy. Also an undoped ZnO epilayer was grown under the conditions mentioned above to compare our results. X-Ray Diffraction (XRD) studies and Secondary Ion Mass Spectrometry (SIMS) studies are carried out to study presence of Bi dopant in the bismuth doped ZnO epilayers. Scanning Electron Micrograph (SEM) and Atomic Force Micrograph (AFM) were also recorded to ensure good surface morphology. Magnetization measurements were carried out using Superconducting Quantum Interface Device (SQUID) magnetometer.

X-ray diffraction measurements of undoped ZnO and $ZnBi_xO_{1-x}$ thin films are presented in **Figure S1**. A dominant (002) peak at ~ 34° and a weak peak at ~ 72° in Figure S1 (a) are observed for both undoped ZnO and $ZnBi_xO_{1-x}$ thin films. X-ray diffraction peaks at 34° and 72° indicate that the thin films are grown along *c*-axis orientation with highly uniform wurtzite

lattice structure. Figure S1(b) shows the magnified scan of the (002) plane in the range of 27° - 40° for undoped ZnO and $ZnBi_{0.01}O_{0.99}$, $ZnBi_{0.03}O_{0.97}$ and $ZnBi_{0.05}O_{0.95}$ thin films. XRD peak positions observed at 33.9°, 34.05° and 34.1° which are shifted compared to undoped ZnO peak positions with the diffraction angle 33.85° clearly manifests the incorporation of Bi in ZnO affects the lattice much along the *c*-axis. Based on these peak positions, the lattice constant *c* was calculated to be 5.29, 5.28, 5.26 and 5.25 Å using Bragg's law for undoped ZnO, $ZnBi_{0.01}O_{0.99}$, $ZnBi_{0.03}O_{0.97}$ and $ZnBi_{0.05}O_{0.95}$, respectively. In comparison with the undoped ZnO film, the $ZnBi_xO_{1-x}$ films show a slight decrease in the lattice constant along the *c*-axis with increasing Bi concentration. The shift in the peak positions and a decrease in the lattice constant value show an opposite trend from the values reported elsewhere[1]. Moreover, β-tetragonal (2θ=27.947°) and α-monoclinic (2θ=28.001°) phases of Bi_2O_3 were not observed for $ZnBi_xO_{1-x}$ films. Hence, it is also evident that there are no secondary phases related to Bi_2O_3.
Secondary Ion Mass Spectrometry (SIMS) measurements were carried out in order to investigate the presence of Bi dopant in the samples. The SIMS profile shows the Bi concentration as a function of film depth in **Figure S2**. It indicates that Bi has essentially been incorporated into ZnO.

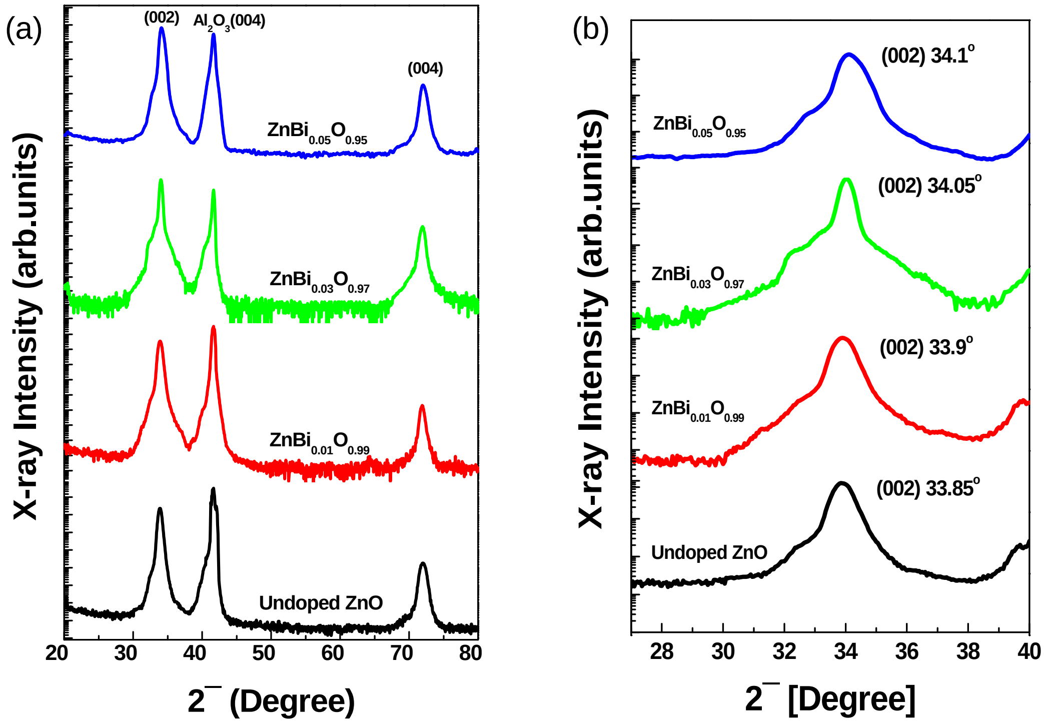

Figure S1. (a) XRD spectrum of undoped ZnO and $ZnBi_{0.01}O_{0.99}$, $ZnBi_{0.03}O_{0.97}$ and $ZnBi_{0.05}O_{0.9}$ thin films. (b) XRD spectrum showing a clear shift in the 2θ values for undoped ZnO versus the $ZnBi_xO_{1-x}$ thin films.

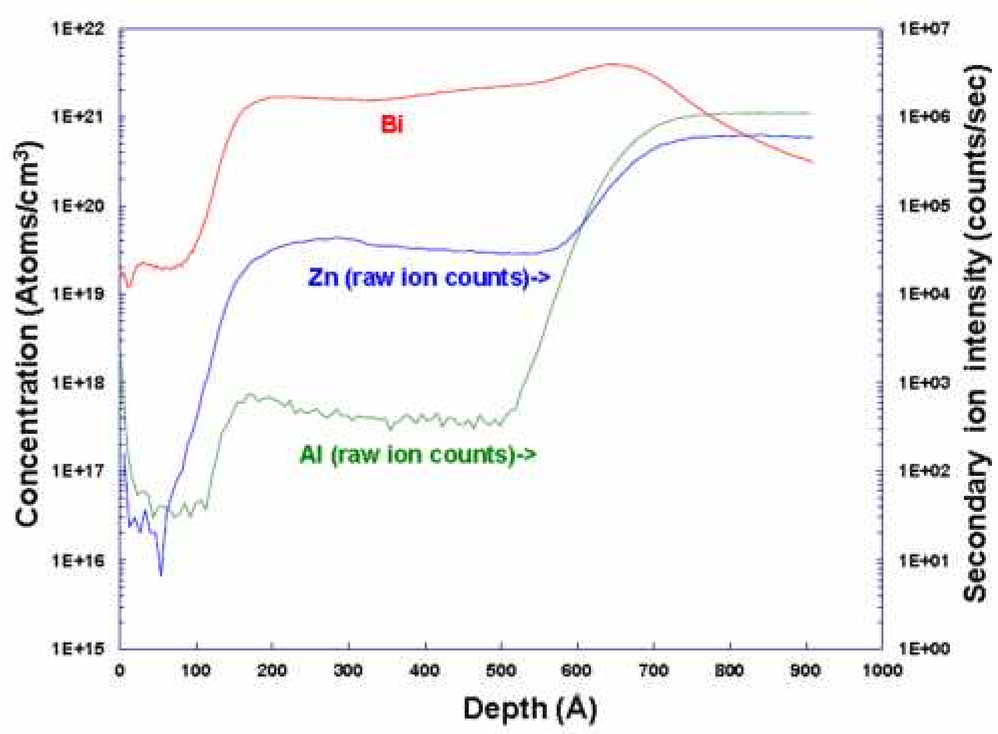

Figure S2. SIMS profiles of a $ZnBi_{0.05}O_{0.95}$ thin film.

Figure S3 shows top view and cross-section SEM images of $ZnBi_xO_{1-x}$ thin films on the (0001) sapphire substrate grown at 600 °C. The top view SEM images indicate that the grown films surface have very uniform surface morphologies without any indications of grains, pinholes. As it can be seen from the images, the obtained $ZnBi_xO_{1-x}$ structures have a good crystalline quality without any columnar structure as seen in cross-section SEM images. It is also evident that these films are single crystalline and this result agrees with the XRD analysis. The film thickness was estimated to be around 200 nm from the cross section SEM images.

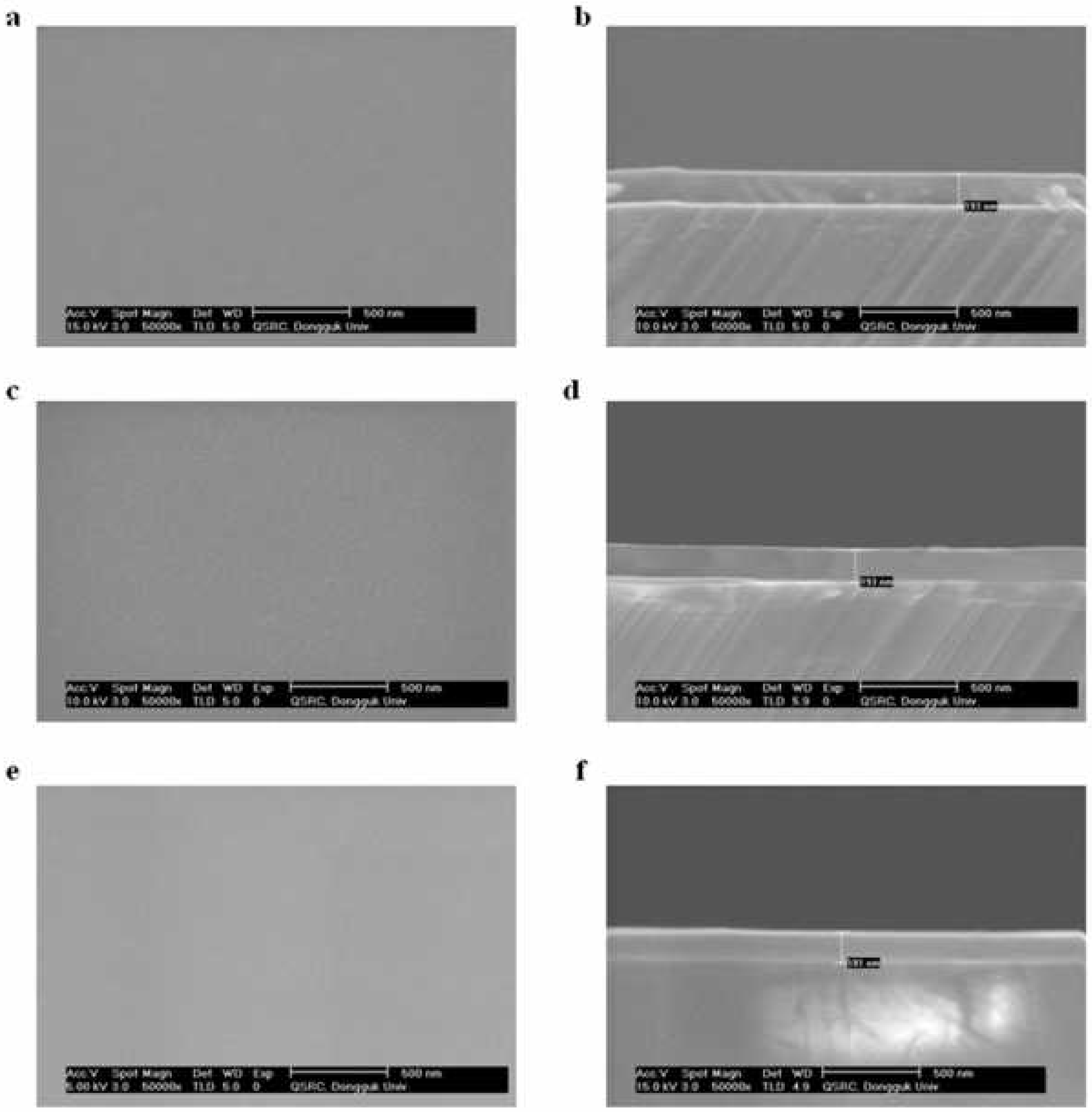

Figure S3. Top view and cross section of SEM images for $ZnBi_xO_{1-x}$ grown on the (0001) sapphire substrate at 600 °C. Panels (a), (c) and (e) show the top view of $ZnBi_{0.01}O_{0.99}$, $ZnBi_{0.03}O_{0.97}$, and $ZnBi_{0.05}O_{0.95}$ thin films. Panels (b), (d) and (f) show the cross sectional view of $ZnBi_{0.01}O_{0.99}$, $ZnBi_{0.03}O_{0.97}$, and $ZnBi_{0.05}O_{0.95}$ thin films, respectively.

Typical 3D-images of $ZnBi_xO_{1-x}$ thin films on (0001) sapphire substrate grown at 1mTorr and 2mTorr oxygen pressure are presented in **Figure S4**. For a scanning area of 3×3 μm^2, very

smooth lateral surface features without any indications of grains and pinholes are seen in the AFM images for $ZnBi_xO_{1-x}$ thin films. As shown in Figure S4, for the thin films grown at 1 mTorr oxygen pressure the maximum value of the Root-Mean-Square (RMS) roughness is 2.042 nm for a $ZnBi_{0.01}O_{0.99}$ thin film and the minimum value is 0.429 nm for a $ZnBi_{0.05}O_{0.95}$ thin film. The average surface roughness (RMS) of all the films was found to be ~ 1.373 nm, independent of the oxygen pressure. As the Bi concentration increases, the surface roughness becomes smaller, which could be due to suppression of oxygen vacancies by bismuth.

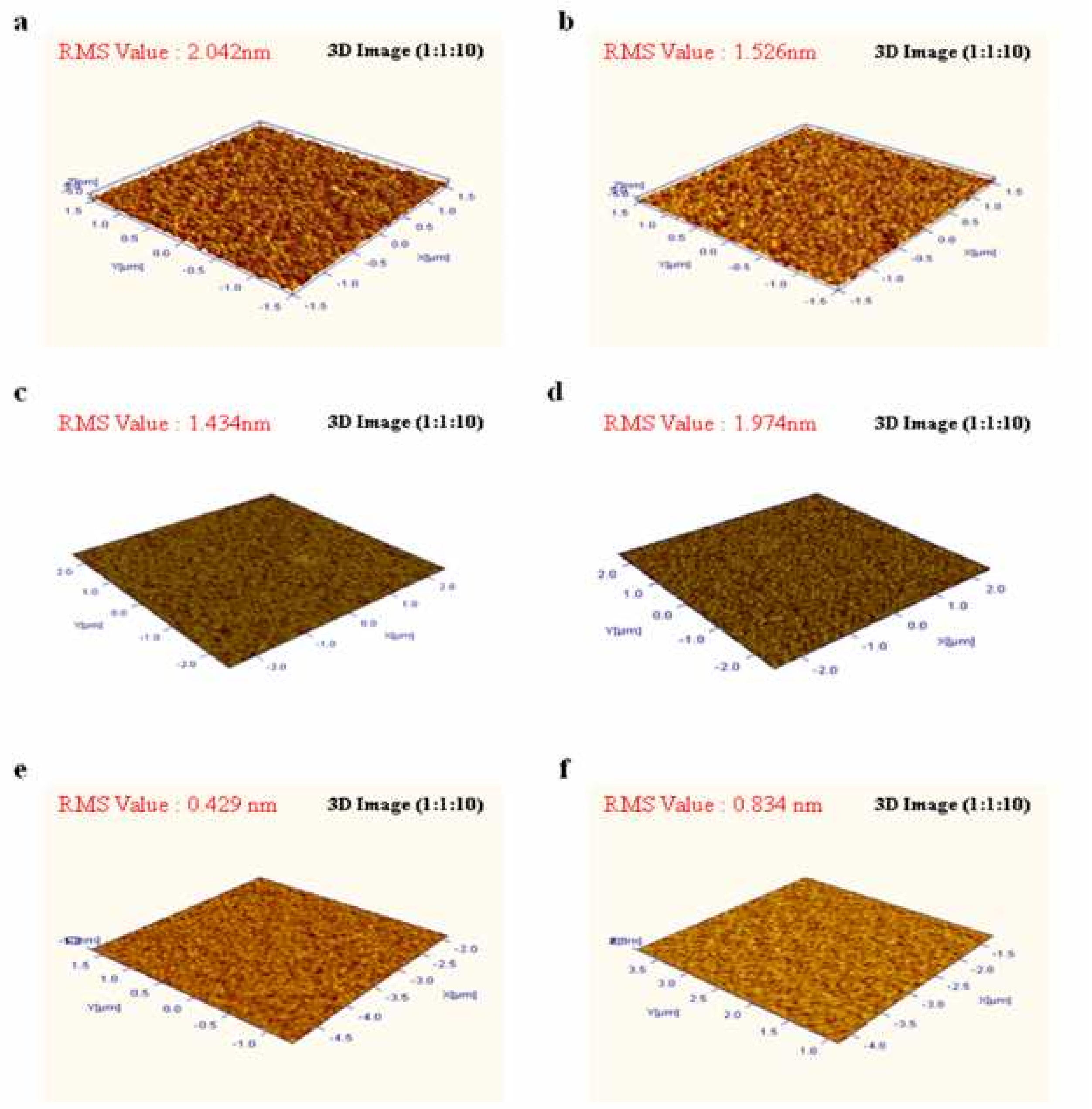

Figure S4. Typical AFM 3D-images of the $ZnBi_xO_{1-x}$ thin films grown on the (0001) sapphire substrate under a 1 mTorr and a 2 mTorr oxygen pressure.

Computational Details:

The generalized gradient approximation (GGA-PBE)[1] is used for the exchange-correlation potential. The plane-wave cutoff energy in our calculations is set to 400 eV. The atomic geometries are fully optimized until the forces on each atom are less than the threshold value of 10^{-4} eV/Å. The valence states of the potentials of Zn, Bi and O are $3d^{10}3p^2$, $6s^26p^3$ and $2s^22p^4$, respectively. The Gaussian smearing width is 0.2 eV. Brillion zone integrations are performed with a Gamma centered 2×2×2 mesh[2]. ZnO has hexagonal wurtzite structure with space group $P6_3mc$ (186) at ambient conditions. The calculated lattice parameters for wurtzite ZnO a = 3.28 Å and c/a = 1.617 within GGA, is in good agreement with experimental data (a = 3.25 Å and c/a = 1.602)[3].

In order to take into account the effects of nonlocal exchange in Bi-doped ZnO, we applied the Heyd-Scuseria-Ernzerhof (HSE) functional[4], which is an approximation of the PBE0 functional [5]. We used the default composition of the PBE0 functional, with 1/4 of exact exchange, E_X and 3/4 of GGA-PBE exchange, E_X^{PBE} , in such way that the functional takes the following form

$$E_{XC}^{PBE0} = \frac{1}{4}E_X + \frac{3}{4}E_X^{PBE} + E_C^{PBE} \quad \text{(S5)}$$

Here, E_C^{PBE} is the PBE correlation energy. We applied the semi-empirical HSE06 functional, where the exact exchange kernel is decomposed onto a local and non-local part and the non-local part is replaced by a Density Functional Approximation part, which is calculated within GGA. In this work, the screening parameter, μ, was set to 0.2, conforming to the HSE06 functional.

[1] J. P. Perdew, Y. Wang, *Phys. Rev. B.* **1992**, *45*, 13244.

[2] H. J. Monkhorst, J. D. Pack, *Phys. Rev. B.* **1976**, *13*, 5188.

[3] R. R. Reeber, *J. Appl. Phys.* **1970**, *41*, 5063.

[4] M. Marsman, J. Paier, A. Stroppa, G. Kresse, *J. Phys. Condens. Matter*. **2008**, *20*, 064201**.**

[5] J. P. Perdew, M. Ernzerhof, K. Burke, *J. Chem. Phys.* **1996**, *105*, 9982.
